# Microbial Diversity of Fermented Greek Table Olives of Halkidiki and Konservolia Varieties from Different Regions as Revealed by Metagenomic Analysis

**DOI:** 10.3390/microorganisms8081241

**Published:** 2020-08-14

**Authors:** Konstantina Argyri, Agapi I. Doulgeraki, Evanthia Manthou, Athena Grounta, Anthoula A. Argyri, George-John E. Nychas, Chrysoula C. Tassou

**Affiliations:** 1 Institute of Technology of Agricultural Products, Hellenic Agricultural Organisation DEMETER, Sofokli Venizelou 1, Lycovrissi, 14123 Athens, Greece; nargiri@gmail.com (K.A.); athenagrounta@gmail.com (A.G.); anthi.argyri@gmail.com (A.A.A.); 2 Laboratory of Food Microbiology and Biotechnology, Department of Food Science and Human Nutrition, School of Food and Nutritional Sciences, Agricultural University of Athens, Iera Odos 75, 11855 Athens, Greece; evita.m@windowslive.com (E.M.); gjn@aua.gr (G.-J.E.N.)

**Keywords:** table olives, Halkidiki olives, Konservolia olives, NGS, Greek-style fermentation, Spanish-style fermentation, microbiological analysis, metagenomic analysis

## Abstract

Current information from conventional microbiological methods on the microbial diversity of table olives is insufficient. Next-generation sequencing (NGS) technologies allow comprehensive analysis of their microbial community, providing microbial identity of table olive varieties and their designation of origin. The purpose of this study was to evaluate the bacterial and yeast diversity of fermented olives of two main Greek varieties collected from different regions—green olives, cv. Halkidiki, from Kavala and Halkidiki and black olives, cv. Konservolia, from Magnesia and Fthiotida—via conventional microbiological methods and NGS. Total viable counts (TVC), lactic acid bacteria (LAB), yeast and molds, and *Enterobacteriaceae* were enumerated. Microbial genomic DNA was directly extracted from the olives’ surface and subjected to NGS for the identification of bacteria and yeast communities. *Lactobacillaceae* was the most abundant family in all samples. In relation to yeast diversity, *Phaffomycetaceae* was the most abundant yeast family in Konservolia olives from the Magnesia region, while *Pichiaceae* dominated the yeast microbiota in Konservolia olives from Fthiotida and in Halkidiki olives from both regions. Further analysis of the data employing multivariate analysis allowed for the first time the discrimination of cv. Konservolia and cv. Halkidiki table olives according to their geographical origin.

## 1. Introduction

Table olives are an important fermented food in Mediterranean countries with great nutritional and economic significance. Their content in bioactive compounds, vitamins, dietary fibers, unsaturated fatty acids, minerals, and antioxidants with demonstrated positive effects on human health meets the consumers’ needs toward natural or minimal processed foods that, beyond basic nutrition, offer additional health benefits [1]. Raised awareness of the health benefits of olives may be partially the driving force for the increased global table olive consumption that has doubled over the past three decades and is expected to increase by 2.1 percent in 2020, as predicted by the International Olive Council (IOC) [2].

In Greece, the table olive industry has evolved in recent years into a dynamic sector of the national economy. With an annual production of 215,000 tons, 85% of which is exported, Greece is the second largest producer of olives in Europe after Spain. The most economically important varieties grown in Greece for table olive processing are Halkidiki and Konservolia, and their final products are sold under the names “green olives Halkidiki variety” and “Greek black olives”, respectively [3].

The Halkidiki variety is primarily grown in the prefecture of Halkidiki, but also in other regions (e.g., Central and Eastern Macedonia). Green olives of the Halkidiki variety have a characteristically large fruit, cylindrical–conical shape, a bright green or greenish-yellow color (it does not turn completely black when it reaches maturity), and outstanding organoleptic characteristics that render the end product a major export product [3]. After harvesting, these olives undergo the Spanish-style processing method by treating the fruit with a diluted NaOH solution (2–3%) to reduce bitterness and also to increase the permeability of olive pericarp. A water wash follows to remove the excess alkali, and olives are then placed in brine (NaCl 8–12%) where the fermentation, driven by lactic acid bacteria, takes place and lasts 3–7 months [4,5].

The Konservolia variety is primarily grown in Central Greece in the prefectures of Fthiotida, Fokida, Magnesia, Aitoloakarnania, Arta, and Evia. Konservolia olives are round to oval in shape, large in size with a high ratio of flesh to pit, and can be transformed into a range of different types of table olives, though the most common type is natural black olives in brine [3]. For this type of preparation, known as Greek-style, olives are immersed directly in a brine solution of about 6–10% NaCl (*w*/*v*) where they undergo natural fermentation for 8–12 months, mainly promoted by yeasts and LAB. The debittering is achieved through the enzymatic activities (mainly β-glucosidase and esterase) of indigenous microorganisms [6,7].

Table olive fermentation processes are a complex microbial ecosystem in which the closely related roles of the LAB and yeast populations are of fundamental importance to obtain high-quality products [8,9]. Currently, two main approaches are adopted to investigate the microbial ecology of table olive fermentations. The culture-dependent techniques that rely on the prior cultivation of the microorganisms are usually applied for the characterization of microbiota present in a specific food ecosystem, however the complete profile of the microbial diversity is underestimated, since they fail to detect populations that are not culturable or in stressed/injured states [10,11]. Recently, culture-independent methods have arisen to overcome the limitations of the classical culture-based approach. The study of microbial diversity is achieved using NGS technologies after direct nucleic acid extraction from the food matrix [12]. Regarding table olive fermentations, culture-independent techniques have been extensively applied in the investigation of the microbial ecology of green olives, belonging mainly to Italian and Spanish varieties, fermented naturally or using the Spanish method [13,14,15,16,17,18]. Furthermore, these studies are usually performed with brines, not taking into consideration the study of the microbial population adhered to olive surface, which is finally the food intake by consumers [19]. However, information about the microbial diversity of natural black and Spanish-style green olive fermentations for Greek table olive varieties is scarce. Recently, Kazou et al. [20] performed 16S and internal transcribed spacer (ITS) metataxonomic analysis to unravel the microbiota of natural black cv. Kalamata fermented olives on both olives and brines.

In the last few years, a large body of scientific research supports the claim that “environment selects”, implying that different contemporary environments maintain distinctive microbial distributions [21]. The idea that free-living microbial taxa exhibit biogeographic patterns was confirmed recently by Lucena-Padros and Ruiz Barba [22], who examined the biogeographic distribution of microorganisms associated to Spanish-style green olive fermentations in the province of Seville. On the other hand, recent studies dispute the idea that “everything is everywhere”, implying that microorganisms have enormous dispersal capabilities that rapidly erase any ecological effects [21]. In this study, the hypothesis that microbial distributions associated with table olive fermentations exhibit biogeographic patterns and therefore differ in different locations was tested by studying table olives from different cultivars originating from different geographical regions. 

The purpose of this study was to assess the microbial diversity of a) Greek-style fermented black olives of Konservolia variety and b) Spanish-style fermented green olives of Halkidiki variety using an NGS approach. The samples were fermented in industrial scale and originated from different geographical regions for each olive variety. Black olives cv. Konservolia were collected from Magnesia and Fthiotida regions and green olives cv. Halkidiki were collected from Halkidiki and Kavala regions. To our knowledge, this is one of the first studies that investigates the microbial ecology of cv. Konservolia and cv. Halkidiki fermented table olives using metagenomic analysis and aims to assess potential biogeographic patterns.

## 2. Materials and Methods 

### 2.1. Olive Sampling

In total, thirty (30) table olive samples—15 samples of fermented cv. Konservolia natural black olives and 15 samples of cv. Halkidiki Spanish-style fermented green olives—were obtained during the 2018–2019 season from two table-olive-producing companies in Greece. The drupes had been collected from four different geographical areas and supplied to the company’s facilities where the fermentations took place according to traditional Greek-style or Spanish-style methods for black and green olives, respectively. Overall, Konservolia variety drupes had been collected from the Magnesia (6 samples) and Fthiotida (9 samples) regions, while Halkidiki variety drupes were collected from the Kavala (6 samples) and Halkidiki (9 samples) regions (Table 1).

### 2.2. Microbiological Analysis

Classical microbiological analysis was performed in olive samples to enumerate the main microbial groups implicated in table olive fermentations [23], i.e., TVC, LAB, yeasts and molds, and *Enterobacteriaceae*. For this purpose, olives were removed from the brine and 25 g of olive flesh was aseptically cut and homogenized in 225 mL sterile ¼ Ringer’s solution (Stomacher 400 circulator, Seward Limited, Norfolk, United Kingdom) for 60 s at room temperature. The appropriate decimal dilutions were poured or spread on the following growth media: (i) Tryptic Soya Agar (TSA, 4021502, Biolife, Milan, Italy ) for TVC enumeration, incubated at 25 °C for 48–72 h; (ii) de Man-Rogosa-Sharpe agar (MRS LAB233, LABM) for the enumeration of LAB, supplemented with 0.05% (*w*/*v*) cycloheximide (AppliChem, Darmstadt, Germany), overlaid with the same medium and incubated at 30 °C for 48–72 h; (iii) Rose Bengal Chloramphenicol Agar (RBC Agar, BK151HA, Biokar diagnostics, Allone, France) for the enumeration of yeasts and molds, incubated at 25 °C for 48 h; and (iii) Violet Red Bile Glucose Agar (VRBGA, CM0485, Oxoid, Hampshire, United Kingdom) for the enumeration of *Enterobacteriaceae*, overlaid with the same medium and incubated at 37 °C for 24 h. The results were log transformed and expressed as log CFU/g.

### 2.3. pH and Salt Measurement

The pH of the brine was recorded using a digital pH meter (Metrohm AG, Herisau, Switzerland). Salt (sodium chloride) determinations in the brines were carried out by titration [24]. The results were expressed as a percentage (*w*/*v*) of NaCl.

### 2.4. Sensory Evaluation

Sensory evaluation of olive samples was performed by a taste panel consisting of ten trained persons according to the method of sensory analysis of table olives established by the IOC [25]. The sensory attributes taken into account included the following descriptors: abnormal fermentation, salty, bitter, acid, hardness, fibrousness, and crispness. Scores were obtained from an evaluation sheet by reading the marks for each descriptor in an unstructured 1–11 scale.

### 2.5. Determination of Olive Microbiota by Next Generation Sequencing (NGS)

For olive microbiota determination, total DNA was directly extracted from olives’ surface using the NucleoSpin Food kit (Macherey-Nagel GmbH & Co. KG, Dueren, Germany) according to the manufacturer instructions. Purified DNA samples were stored at −20 °C until use. 

The Ion 16S Metagenomics kit (Thermo Fisher Scientific, Waltham, MA, USA) was used to amplify the V2-4-8 and V3-7-9 hypervariable regions of 16S rRNA gene, and the resulting amplicons (400 bp) were sequenced using Ion Torrent PGM by CeMIA SA (https://cemia.eu/) (Larissa, Greece) to estimate the bacterial diversity. The analysis of sequences was performed using Ion Reporter software (Thermo Fisher Scientific, Waltham, MA, USA). Chimeras and noise were removed from the sequences. Operational taxonomic units (OTUs) were taxonomically classified (at >97% similarity) using Nucleotide Basic Local Alignment Search Tool (BLASTn) against the NCBI database (www.ncbi.nlm.nih.gov) (Bethesda MD, 20894 USA).

For yeast/fungal ecology estimation, amplicon sequencing (bTEFAP) was performed on the Illumina MiSeq at Molecular Research DNA (MR DNA, Shallowater, Texas). The ITS primer pair, ITS1F (5ʹ-CTTGGTCATTTAGAGGAAGTAA-3ʹ) and ITS2R (5ʹ-GCTGCGTTCTTCATCGATGC-3ʹ), was used to amplify the yeast/fungal internal transcribed spacer (ITS) DNA region, namely, ITS1-ITS2. Each sample underwent a single-step 30 cycle PCR using HotStarTaq Plus Master Mix Kit (Qiagen, Valencia, CA, USA). Following PCR, and all amplicon products from different samples were mixed in equal concentrations and purified using Agencourt Ampure beads (Agencourt Bioscience Corporation, Beverly, MA, USA). Samples were sequenced utilizing the Illumina MiSeq chemistry following the manufacturer’s protocols. Sequences were then denoised and chimeras removed. Operational taxonomic units were defined after removal of singleton sequences, clustered at 3% divergence (97% similarity) and taxonomically classified using BLASTn against a curated NCBI deriving database [26] and compiled into each taxonomic level as percentages, reflecting the relative percentage of sequences within each sample.

Microbial diversity was analyzed using the R package Phyloseq v. 3.6.1. [27]. OTU abundance was normalized using the median sequencing depth of all samples. Analyses of alpha diversity were carried out using standard or custom Phyloseq command lines.

### 2.6. Statistics and Multivariate Analysis

Differences in microbial populations, tested with one-way analysis of variance (ANOVA) followed by post hoc comparisons with Tukey’s test, were considered statistically significant at *p* < 0.05. Analysis of data was carried out with Statistica 8.0 software package (StatSoft Inc., Tulsa, OK, USA).

Partial least squares discriminant analysis (PLS-DA), was used to optimize separation between the different olive samples by linking two data matrices *X* (i.e., raw data) and *Y* (i.e., classes) [28]. In our case, the raw data used were the microbiological, physicochemical, sensory data as well as the characterized microbiota (bacteria and yeasts) at species level OTUs. The tested classes were either the cultivars (i.e., Konservolia and Halkidiki) or the four geographical sampling regions (i.e., Magnesia, Fthiotida, Halkidiki and Kavala). Data were transformed by autoscaling before analysis. In addition, the variable importance on projection (VIP) was used to identify the most important variables. A VIP value of 1.0 has generally been accepted as a cut-off limit in variable selection; thus, variables exceeding this limit were considered to be highly influential [20]. Heatmaps were also performed for data visualization. PLS-DA analysis and heatmaps were performed using MetaboAnalyst 4.0 [29].

## 3. Results

### 3.1. Microbial and Physicochemical Quality of Fermented Table Olives

**Figure 1:**
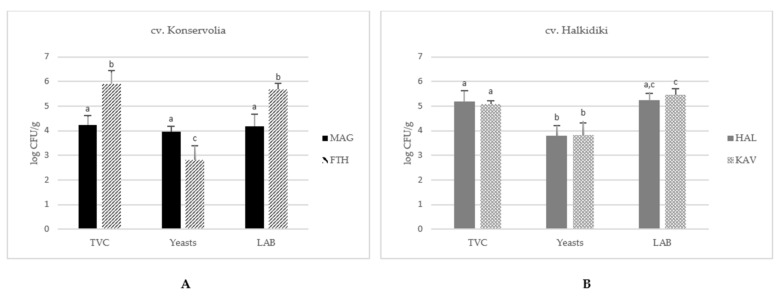
Microbial enumerations of total viable counts (TVC), lactic acid bacteria (LAB), and yeasts in fermented table olives of (**A**) cv. Konservolia from Magnesia (MAG) and Fthiotida (FTH) regions and (**B**) cv. Halkidiki from Kavala (KAV) and Halkidiki (HAL) regions. The results present average values ± SD. Different letters indicate statistically significant differences (*p* < 0.05).

Figure 1 illustrates the mean population of TVC, LAB, and yeasts and molds in fermented table olives of Konservolia cultivar from the Magnesia and Fthiotida regions (Figure 1A) and Halkidiki cultivar from the Halkidiki and Kavala regions (Figure 1B). The specific population of the different microbial groups enumerated on each sample is shown at Table S1. TVC in natural fermented table olives of Konservolia variety exhibited an average value of 5.2 ± 0.9 log CFU/g, with the counts of olives from the Fthiotida region exhibiting about 2-log units higher value than the corresponding average population in olives harvested from Magnesia (*p* < 0.05) (Figure 1A). Similarly, the LAB population exhibited average values of 5.7 ± 0.2 log CFU/g and 4.2 ± 0.5 log CFU/g in table olives from the Fthiotida and Magnesia regions, respectively. By contrast, the yeast population in table olives from the Fthiotida was 2.8 ± 0.5 log CFU/g, about 1-log unit lower than the respective population in olives from the Magnesia region (*p* < 0.05).

In the case of Spanish-style fermented green olives of Halkidiki variety, no significant differences were observed in the microbial populations between samples from the Halkidiki and Kavala regions (*p* > 0.05) (Figure 1B). It has to be noted that, in all samples, *Enterobacteriaceae* population was lower than the detection limit of the method (<1 log CFU/g). 

Regarding pH measurements, the pH values in the brine of olives did not exceed 4.30 (Table S1), a finding complying with the physicochemical characteristics for the safety of the final product [30]. More specifically, the pH value of brine samples from Magnesia (3.58 ± 0.01) was significantly lower (*p* < 0.05) than the pH of brine samples from the Fthiotida region (4.14 ± 0.14). On the other hand, brine samples from the Kavala and Halkidiki regions presented average similar pH values, i.e., 3.81 ± 0.04 and 3.79 ± 0.01, respectively. Moreover, the salt concentration in the brines of samples from Magnesia (10.3 ± 0.17% *w*/*v*) was significantly higher than the salt concentration in brine samples from Fthiotida (5.45 ± 0.78% *w*/*v*) (*p* < 0.05). Similarly, significant differences were also observed in salt concentration between brine samples from Kavala (5.75 ± 2.01% *w*/*v*) and Halkidiki (7.63 ± 0.13% *w*/*v*) (*p* < 0.05).

### 3.2. Sensory Evaluation of Fermented Table Olives

**Figure 2:**
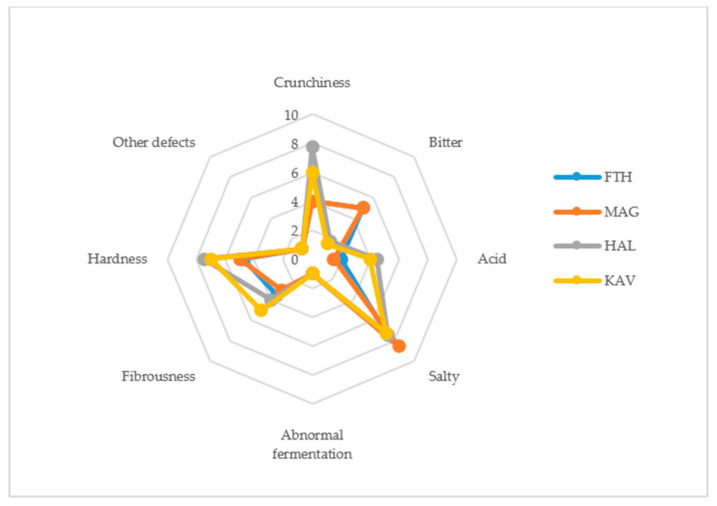
Spider graph showing the sensory profiles (original scores) for the diverse fermented table olives samples. FTH (origin, Fthiotida; cultivar, Konservolia), MAG (Magnesia; Konservolia), HAL (Halkidiki; Halkidiki), KAV (Kavala; Halkidiki).

The scores of sensory attributes evaluated for the fermented table olives are presented in Figure 2. Regarding the gustatory sensations, naturally fermented black olives of cv. Konservolia from Fthiotida and Magnesia were perceived to be bitterer than green olives of cv. Halkidiki. Black olives from Magnesia received the highest score for saltiness by the panelists. Concerning acidity, green olives from Halkidiki and Kavala received higher scores compared to black olives from Fthiotida and Magnesia that developed a milder taste. No off odors indicating abnormal fermentation (i.e., butyric, putrid fermentation or zapateria) or other defects were detected by the panelists in any of the table olive samples. Moreover, Spanish-style fermented green olives of cv. Halkidiki from Halkidiki and Kavala received higher scores for the kinesthetic sensations of hardness, fibrousness, and crunchiness compared to naturally fermented black olives of cv. Konservolia from Fthiotida and Magnesia.

### 3.3. Bacterial Community Profiling

**Figure 3:**
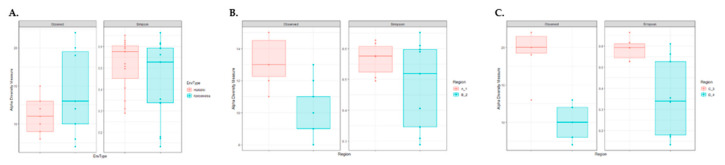
Alpha-diversity boxplots for table olive’s bacterial families of (**A**) cultivar Halkidiki and Konservolia, (**B**) cultivar Halkidiki from Halkidiki (A_1) and Kavala (B_2) regions, and (**C**) cultivar Konservolia from Magnesia (C_3) and Fthiotida (C_4) regions based on observed and Simpson indices.

The NGS of 16S rRNA amplified from total DNA extracted from the surface of olive samples was applied to monitor the bacterial relative abundancies. The metataxonomic analysis in cv. Konservolia and cv. Halkidiki table olives revealed a complex bacterial microbiota that consisted of thirteen and eleven families, respectively. In brief, differences in bacterial families (Figure 3A) and species (data not shown) were observed based on olive varieties and were significantly higher (*p* < 0.05) in cv. Halkidiki than in cv. Konservolia table olives. In the case of cv. Halkidiki table olives, bacterial families were significantly higher in olives from the Halkidiki region than from the Kavala region (*p* < 0.05) (Figure 3B). Similarly, significantly higher bacterial communities were observed in table olives from the Magnesia region than from the Fthiotida region (Figure 3C).

**Figure 4:**
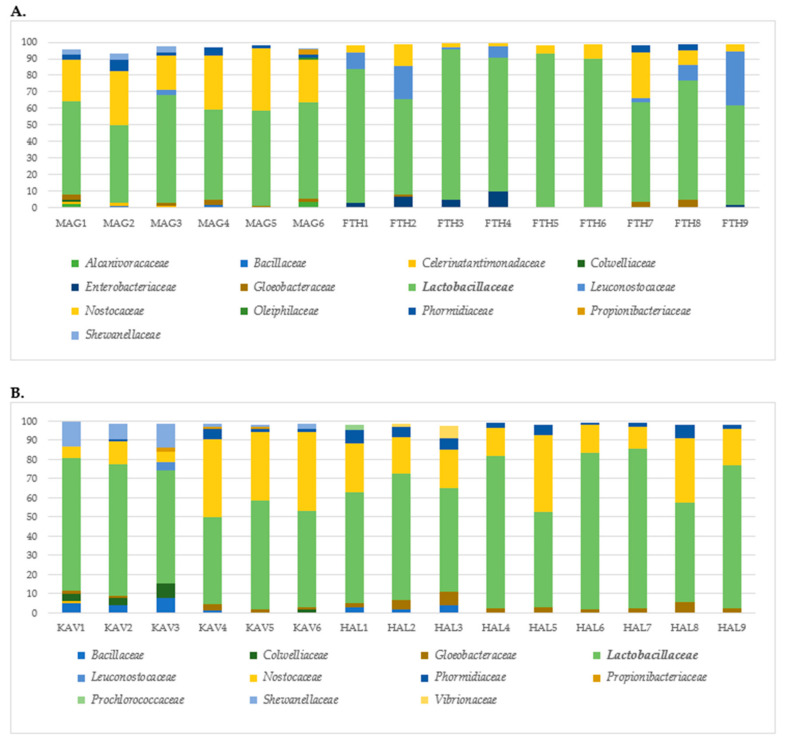
Relative abundance of total observed bacterial families on table olives of (**A**) cv. Konservolia originating from the regions of Magnesia (MAG) and Fthiotida (FTH) and (**B**) cv. Halkidiki originating from the regions of Kavala (KAV) and Halkidiki (HAL). Only families above 1% occurrence are reported.

In Figure 4, the OTUs at family level on the olive surface of cv. Konservolia (Figure 4A) and cv. Halkidiki (Figure 4B) samples, representing at least 1% of the total sequence reads in each sample, are displayed. *Lactobacillaceae* was the predominant bacterial family identified across all olive samples of cv. Konservolia and cv. Halkidiki from both geographical regions (Figure 4A,B). The whole set of identifications at family, genus, and species level is shown as Supplementary Material (Table S2A–C).

*Lactobacillus* was the most common detected genus in all cases, followed by *Pediococcus* in samples of cv. Konservolia and samples from the Halkidiki region. In brief, the species *Lactobacillus acidipiscis*, *Lactobacillus coryniformis*, *Lactobacillus paracollinoides*, *Lactobacillus parafarraginis*, *Lactobacillus harbinensis*, *Lactobacillus kisonensis*, *Pediococcus parvulus*, and *Pediococcus ethanolidurans* were identified (Table S2C). Furthermore, *Nostocaceae* was the second most common family found in samples from Magnesia, Kavala and Halkidiki, whereas *Leuconostocaceae* was the second abundant family detected in samples from the Fthiotida region. Concerning the rest of the detected bacteria, in olives from Magnesia, *Shewanellaceae* (including *Shewanella*), *Propionibacteriaceae* (including *Propionibacterium*), and *Gloeobacteraceae* were also detected, with the remaining families being present at lower proportions (<2%) (Table S2B). Similarly, *Nostocaceae*, *Enterobacteriaceae*, *Gloeobacteraceae*, and *Phormidiaceae* were in samples from the Fthiotida region (Figure 4A). Moreover, other families contributing to the bacterial consortium in olives from Kavala were *Shewanellaceae* (including *Shewanella*), *Bacillaceae*, *Colwelliaceae*, *Gloeobacteraceae*, and *Propionibacteriaceae*, while *Leuconostocaceae* was identified only in one sample (Figure 4B). On the other hand, *Phormidiaceae*, *Vibrionaceae*, *Gloeobacteraceae*, *Prochlorococaceae*, and *Bacillaceae* were also detected on table olives from the Halkidiki region (Figure 4B).

### 3.4. Yeast Community Profiling

**Figure 5:**
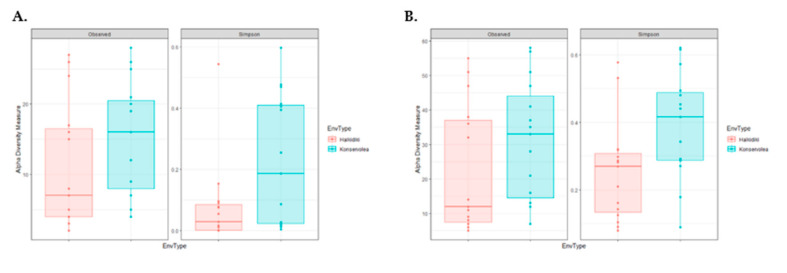
Alpha-diversity boxplots for table olives yeasts families (**A**) and species (**B**) of cultivar Halkidiki and Konservolia based on observed and Simpson indices.

**Figure 6:**
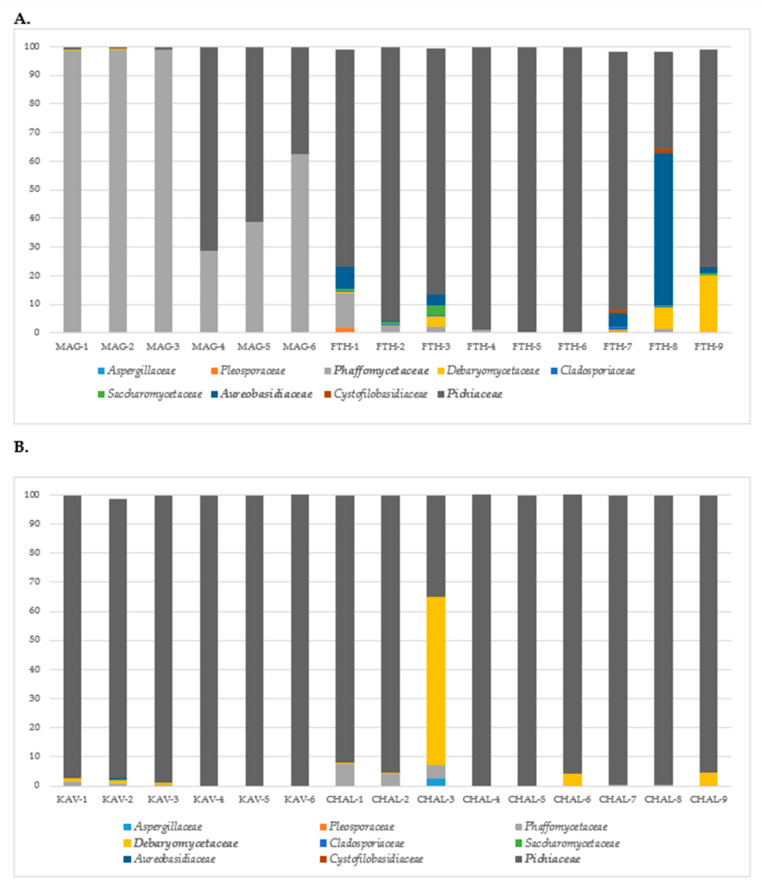
Relative abundance of total observed yeast families on table olives of (**A**) cv. Konservolia originating from the regions of Magnesia (MAG) and Fthiotida (FTH) and (**B**) cv. Halkidiki originating from the regions of Kavala (KAV) and Halkidiki (HAL). Only families above 1% occurrence are reported.

The yeast community of olive samples was revealed by NGS of the ITS region of yeast rDNA amplified from total DNA extracted from the surface of fermented table olive samples. The metataxonomic analysis in cv. Konservolia and cv. Halkidiki table olives revealed a complex yeast microbiota. In brief, differences in yeast families and genera were observed based on olive varieties and were significantly higher (*p* < 0.05) in cv. Konservolia than cv. Halkidiki table olives (Figure 5). However, no significant differences were observed between the different geographical regions for both cultivars (data not shown). The detected families at relative abundance >1% of the total sequence reads in each olive sample are presented in Figure 6, while the whole set of identifications at family, genus, and species level is shown as Supplementary Material (Table S3A–C).

*Pichiaceae* was mainly detected at highest relative abundance in green, Spanish-style fermented olives cv. Halkidiki from both geographical regions (Figure 6B) and the majority of olive samples cv. Konservolia (Figure 6A). In the case of Konservolia olives from Magnesia, *Phaffomycetaceae* was the dominant family in four samples, followed by *Pichiaceae* that dominated in two samples, while the remaining families were present at very low proportions (<1%) (Figure 6A). In the latter case, the most detected species were *Wickerhamomyces anomalus*, *Pichia membranifaciens*, and *Wickerhamomyces sydowiorum* (Table S3C). Similarly, *Pichiaceae* was the predominant family across eight out of nine samples, followed by *Aureobasidiaceae* that dominated the yeast community in one sample in olives from Fthiotida. The rest of the families, i.e., *Debaryomycetaceae* and *Phaffomycetaceae*, were present at very low proportions (<1%) (Figure 6A). In brief, *Pichia manshurica*, *Brettanomyces custersianus*, *Pichia membranifaciens*, *Aureobasidium pullulans*, *Schwanniomyces etchelsii*, and *Wickerhamomyces anomalus* were characterized at species level (Table S3C). On the other hand, for cv. Halkidiki olives from Kavala, beyond *Pichiaceae* which was the most detected family, the rest of the families were detected in low relative percentages (< 1%) (Figure 6B). In brief, the yeast microbiota was dominated by *Pichia* (including *Pichia manshurica*) and *Brettanomyces* (including *Brettanomyces custersianus*) (Table S3B,C). In the case of olives from the Halkidiki region, the microbiota of one sample out of six was dominated by *Debaryomycetaceae*, while the majority of them were dominated by *Pichiaceae*. *Pichia* (including *Pichia manshurica* and *Pichia membranifaciens*) was the dominant genus detected in eight out of nine samples, while *Schwanniomyces* (i.e., *Schwanniomyces etchelsii*) dominated the ninth sample followed by *Ogataea*, *Pichia*, and *Penicillium* (Table S3B,C).

### 3.5. Cultivar and Geographical Discrimination of Table Olives by Multivariate Analysis

**Figure 7:**
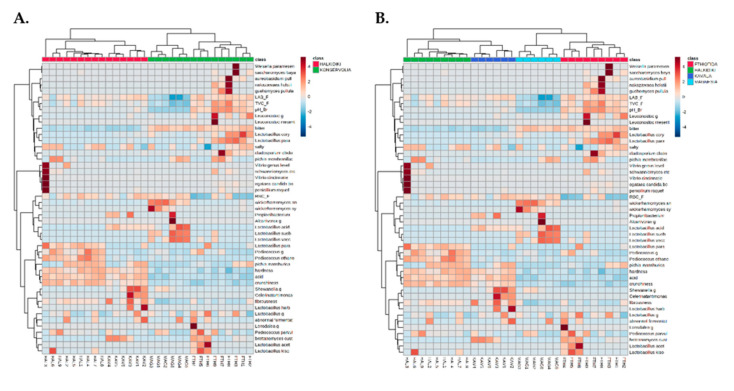
Hierarchically clustered heatmap of microbiological, physicochemical, organoleptic, and species level operational taxonomic units (OTUs) of bacteria and yeast communities data of table olive samples based on (**A**) the cultivar and (**B**) the geographical origin of the samples. The sample codes are indicated in Table 1.

A dual hierarchal dendrogram (heatmap) was utilized to display the data obtained from this study (microbiological, physicochemical, sensory, and bacterial and yeast species—level OTUs) with clustering related to the different olive samples. Based on the clustering evident in Figure 7, there appears to be a clear distinction between samples based on cultivar (Figure 7A) and geographical origin (Figure 7B) classes. 

**Figure 8:**
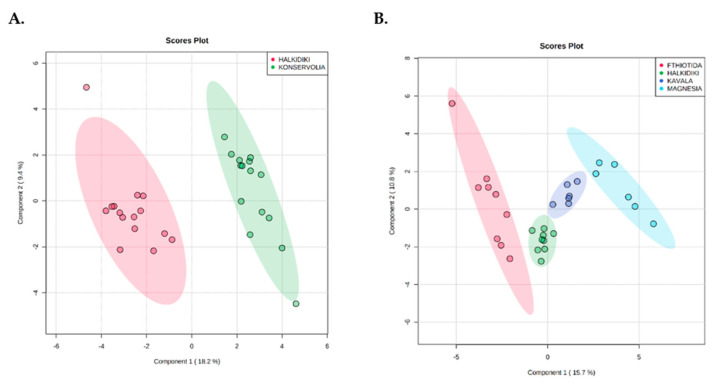
Partial least squares discriminant analysis (PLS-DA) clustering depending on (**A**) cultivar and (**B**) geographical origin of the olive samples.

**Figure 9:**
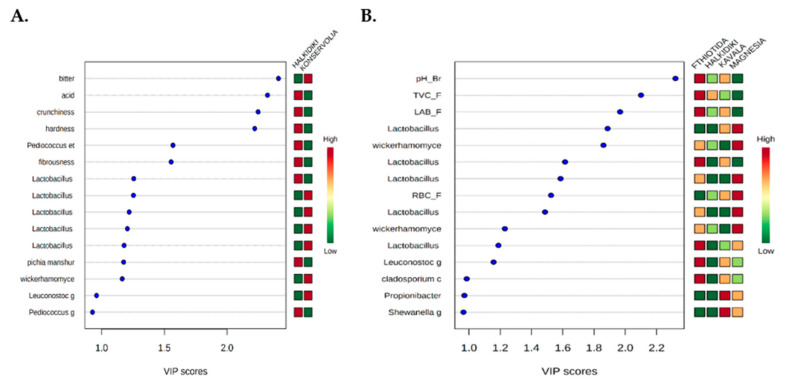
Most influential parameters of the olive samples based on the VIP scores from the PLS-DA analysis at (**A**) cultivar and (**B**) geographical origin levels.

Furthermore, PLS-DA analysis effectively discriminated olive samples based on cultivar (Figure 8A) and geographical origin (Figure 8B) classes with no overlapping. However, a statistically significant difference (*p* < 0.001) was confirmed only for the discrimination of olives based on geographical origin. In this case, according to the VIP values (>1), pH_Br, TVC-F and LAB_F and *Lactobacillus paracollinoides*, *Lactobacillus coryniformis*, *Leuconostoc*, and *Cladosporium cladosporioides* were highly associated with the Fthiotida region (Figure 9B). Similarly, *Lactobacillus acidipiscis*, *Wickerhamomyces anomalus*, *Lactobacillus suebicus*, RBC_F, *Lactobacillus vaccinostercus*, and *Wickerhamomyces sydowiorum* were highly associated (VIP value > 1) with the Magnesia region and *Propionibacterium* and *Shewanella* with the Kavala region (Figure 9B).

## 4. Discussion

The effect of cultivar and geographical origin on the microbiota of the fermented table olives was assessed in this research. For this purpose, the bacterial and yeast diversity of fermented table olives of two main Greek varieties collected from different regions, i.e., black olives, cv. Konservolia, from Magnesia and Fthiotida and green olives, cv. Halkidiki, from Kavala and Halkidiki was evaluated using metataxonomics in parallel with the classical microbiological approach and taking into account physicochemical and organoleptic characteristics. The characterization of the microbial communities of Greek table olives aims at a comprehensive analysis of their microbial ecology and contributes to the exploitation of their microbial fingerprint based on cultivar and area of origin.

PLS-DA analysis indicated a satisfactory discrimination among the different geographical regions without overlapping between the cases, with the pH value and the TVC and LAB counts representing the most discriminative parameters. 

LAB was the predominant microbial population in black Greek-style fermented olives cv. Konservolia from Fthiotida and in green Spanish-style fermented olives cv. Halkidiki from both geographical regions. The dominance of LAB is rather typical for Spanish-style processing and has been previously observed by other researchers [15,31]. This observation is also in line with previous findings for Greek table olives and is attributed to the low salt level (6–7%) used by the Greek industry in the brine during the period of active fermentation to ensure the dominance of LAB and therefore improve the preservation and sensory characteristics of the final product [6,32]. It is well documented that yeast development is favored against LAB by high salt concentrations, the presence of phenolic compounds, and low pH levels [33,34,35]. Low pH values and high salt concentrations were also measured in the case of black olives from the Magnesia region, where LAB and yeasts were detected at similar levels. The involvement of yeasts is particularly important in natural olives, when fruits are not lye-treated and phenolic compounds partly inhibit LAB development [36]. Similar yeast populations, i.e., 4.7 log cfu/g and 4 log cfu/mL were previously enumerated in Greek black dry-salted olives (cv. Thassos) with ~7.5% NaCl [37] and black table olives of cv. Hojiblanca with 4% NaCl and 0.3% acetic acid [38], respectively. 

The metataxonomic analysis employed herein highlighted differences in bacterial and yeast ecology both at cultivar and geographical origin levels. *Lactobacillaceae* was the dominant family identified in olive samples from both cultivars, indicating that these were all lactic acid fermentations, which was also verified by the classical microbiological analysis. The significant role of this microbial group in olive fermentations has been extensively reviewed by Hurtado et al. [39], and it is commonly found in the microbiota of fermented green and black olives using both classical microbiological and metagenomics analyses [7,15,18,40,41,42]. NGS highlighted relevant differences in the occurrence of different *Lactobacillus* species, depending on the cultivar. According to multivariate analysis, the most discriminative species were *Lactobacillus acidipiscis*, *Wickerhamomyces anomalus*, and *Lactobacillus paracollinoides* (VIP > 1.6). In a recent study, the presence of *Lactobacillus* was also highly influential for the differentiation of Greek-style fermented olives cv. Kalamata from different geographic regions [20]. Furthermore, the species *Lactobacillus paracollinoides* was identified as responsible for the discrimination of Spanish-style green olive fermentations among different patios [22]. Moreover, *L. harbinensis* was found to colonize only the surface of green Spanish-style fermented table olives cv. Halkidiki, while *L. vaccinostercus*/*L. suebicus*, described by Abriouel et al. [13] were detected only on the surface of black naturally fermented table olives cv. Konservolia underlining the impact of cultivar in microbial diversity. In the present study, the occurrence of *L. harbinensis* at fermented table olives was revealed for the first time. *L. harbinensis* is a halotolerant species often isolated from fermented vegetables and dairy products [43]. However, the detection of *L. coryniformis* has also been reported previously in green table olive fermentations [44,45] and in black olives packed in modified atmosphere conditions [46]. In addition, *L. acidipiscis* was detected in green olives cv. Halkidiki from the Kavala region and in black olives cv. Konservolia from the Magnesia region, reinforcing the importance of regional characteristics (e.g., climatic conditions) in microbial diversity. Likewise, *L. paracollinoides* was detected in black table olives from Fthiotida and in one sample of green olives from Kavala. The occurrence of *L. paracollinoides* in table olive fermentations has been reported previously [13,22]. Moreover, pediococci were also detected in olives of both cultivars, with a higher abundance in green olives from Halkidiki where in some samples, they dominated over the *Lactobacillus* population. The dominant species were *Pediococcus parvulus*, detected in olives from both cultivars and *Pediococcus ethanolidurans* found in higher abundance in olives from Halkidiki than Konservolia variety, as it was detected at low relative abundance only in olives from Magnesia. In earlier studies, *Pediococcus ethanolidurans* was also isolated from black [46] and green [16] olive fermentations, while *P. parvulus* was found to be the dominant species in green table olives [47]. Regarding the rest of the LAB, the high relative abundance of *Leuconostoc* genus in black olives cv. Konservolia from the Fthiotida region in combination with the low salt concentration of these samples is in accordance with previous findings that observed a high occurrence of these heterofermentative cocci in fermentations carried out in brine with a low salt concentration [45].

An unusual finding of the present study was the detection of cyanobacteria in the microbiota of fermented table olives of Konservolia and Halkidiki cultivars, represented mainly by *Nostocaceae* family, followed by *Phormidiaceae* and *Gloeobacteraceae* with relative lower abundances. Cyanobacteria are ubiquitously present in soil and marine environments, and some species can survive harsh environmental conditions, including environments with high salt concentrations [48]. Their presence has been highlighted in earlier studies conducted on table olives [49] and olive-mill wastewater [50]; however, it should be carefully evaluated due to emerging human health issues related to this bacterial group [51,52,53]. 

Moreover, *Enterobacteriaceae* was detected in black naturally fermented olives cv. Konservolia only in some samples from the Fthiotida region at low relative abundances, although its presence was not confirmed by the classical microbiological methods. This could be attributed either to the amplification of DNA from dead bacteria or to the low detection limit of the plate counting method [49]. The presence of this family in the fermentation of table olives is rather habitual, with a well-known negative contribution in the quality of the final product [54].

Similarly, yeast diversity on olive surfaces was determined by targeting the ITS region of the nuclear ribosomal DNA, a widely accepted standard procedure for yeast identification not only in fermented table olives [19,20] but also in other food fermentations [55]. According to the results, the yeast microbiota of olive samples of both cultivars was less diverse compared to bacteria, a finding in accordance with the results obtained previously regarding fermented natural black olives cv. Kalamata [20].

*Pichiaceae* was the dominant family identified in green olives cv. Halkidiki from both regions, confirming its ability to colonize the surface of table olives [8]. Specifically, green olives from Kavala showed a homogeneous yeast population where *Pichiaceae* family prevailed in all samples. The species *Pichia manshurica, Brettanomyces custersianus, Pichia membranifaciens, Schwanniomyces etchelsii*, and *Ogataea candida boidinii* were the most common species detected. These results are in agreement with a recent work, where *Pichia manshurica*, *Pichia membranifaciens*, and *Schwanniomyces etchellsii* were found among the yeast species at the final stage of Spanish-style green olive fermentation [22]. The low occurrence (<1%) of *Saccharomyces* in the observed yeast consortium is of importance, as this genus has been highly associated with olive fermentation [9]. This finding is consistent with the results of biofilm community formed on the surface of plastic vessels used in Spanish-style green olive fermentation cv. Halkidiki [56] and middle stage of Spanish-style fermentation [22]. On the other hand, *Brettanomyces* are usually associated with the fermentation of alcoholic beverages like beer and wine having a controversial role from spoilage organisms to contributors to industrial fermentations. However, *Brettanomyces* was also recently detected in black olives cv. Kalamata at low levels [20], while *Brettanomyces custersianus* and *D. bruxellensis* have been isolated in the past from olives [57] and Greek-style black olives [58], respectively. It has to be noted that differences were observed among the dominant yeast families in black natural olives cv. Konservolia between the samples from the different geographical regions. The dominant yeast families identified in samples from Magnesia were *Phaffomycetaceae* (mainly *Wickerhamomyces anomalus*), followed by *Pichiaceae* (mainly *Pichia membranifaciens*). On the other hand, *Pichiaceae* was identified as the dominant yeast family in most of the black natural olives cv. Konservolia from the Fthiotida region, followed by *Aureobasidiaceae*. The prevalence of *W. anomalus* in the yeast consortium was probably attributed to its tolerance to diverse stress factors such as low pH and high salt concentration, characteristics found in the brines of the samples from Magnesia. Earlier studies have confirmed its presence in natural black olives of cv. Konservolia [59,60]. *W. anomalus* has been reported to exhibit β-glucosidase activity and produce antioxidant compounds and killer toxins against human pathogens and spoilage microorganisms [61,62], properties that may improve the quality of the final product both from nutritional and safety aspects. *Pichia manshurica*, *Brettanomyces*, and *Pichia membranifaciens* were also isolated recently from natural black olive fermentations of Konservolia and Kalamata cultivars [20,59,60], while *Aureobasidium pullulans* has been previously detected on first stages of cv. Konservolia olive fermentation [59] and Kalamata black olive natural fermentations [63]. *P. membranifaciens* has shown strain-specific killer activity against spoilage yeasts, thus preventing food spoilage [64].

## 5. Conclusions

In conclusion, discriminative analysis was performed to detect biogeographic patterns of the microbial populations along with physicochemical and organoleptic characteristics of Greek fermented table olives belonging to Konservolia and Halkidiki varieties. The diversity of the microbial community of olives from different regions was evaluated by metataxonomic analysis. The results obtained reveal the complex structure of the microbiota in these fermentations and point the microbial key taxa that may be linked to specific geographic areas. However, further studies are needed to enhance our knowledge of the microbial ecology of Greek table olives and probably enable the design of new strategies to improve their quality and safety.

## Figures and Tables

**Table 1:** Geographical origin of the fermented cv. Konservolia natural black and cv. Halkidiki Spanish-style green olive samples.

Samples	Variety	Region	Origin	Olive Colour	Fermentation Type
MAG1	Konservolia	Central Greece	Magnesia	Black	Greek-style
MAG2	Konservolia	Central Greece	Magnesia	Black	Greek-style
MAG3	Konservolia	Central Greece	Magnesia	Black	Greek-style
MAG4	Konservolia	Central Greece	Magnesia	Black	Greek-style
MAG5	Konservolia	Central Greece	Magnesia	Black	Greek-style
MAG6	Konservolia	Central Greece	Magnesia	Black	Greek-style
FTH1	Konservolia	Central Greece	Fthiotida	Black	Greek-style
FTH2	Konservolia	Central Greece	Fthiotida	Black	Greek-style
FTH3	Konservolia	Central Greece	Fthiotida	Black	Greek-style
FTH4	Konservolia	Central Greece	Fthiotida	Black	Greek-style
FTH5	Konservolia	Central Greece	Fthiotida	Black	Greek-style
FTH6	Konservolia	Central Greece	Fthiotida	Black	Greek-style
FTH7	Konservolia	Central Greece	Fthiotida	Black	Greek-style
FTH8	Konservolia	Central Greece	Fthiotida	Black	Greek-style
FTH9	Konservolia	Central Greece	Fthiotida	Black	Greek-style
KAV1	Halkidiki	Macedonia	Kavala	Green	Spanish-style
KAV2	Halkidiki	Macedonia	Kavala	Green	Spanish-style
KAV3	Halkidiki	Macedonia	Kavala	Green	Spanish-style
KAV4	Halkidiki	Macedonia	Kavala	Green	Spanish-style
KAV5	Halkidiki	Macedonia	Kavala	Green	Spanish-style
KAV6	Halkidiki	Macedonia	Kavala	Green	Spanish-style
HAL1	Halkidiki	Macedonia	Halkidiki	Green	Spanish-style
HAL2	Halkidiki	Macedonia	Halkidiki	Green	Spanish-style
HAL3	Halkidiki	Macedonia	Halkidiki	Green	Spanish-style
HAL4	Halkidiki	Macedonia	Halkidiki	Green	Spanish-style
HAL5	Halkidiki	Macedonia	Halkidiki	Green	Spanish-style
HAL6	Halkidiki	Macedonia	Halkidiki	Green	Spanish-style
HAL7	Halkidiki	Macedonia	Halkidiki	Green	Spanish-style
HAL8	Halkidiki	Macedonia	Halkidiki	Green	Spanish-style
HAL9	Halkidiki	Macedonia	Halkidiki	Green	Spanish-style

## Supplementary Materials

The following are available online at https://www.mdpi.com/2076-2607/8/8/1241/s1, Table S1: Microbiological counts of TVC, yeasts, LAB, and *Enterobacteriaceae* from olives (O) and pH and salt concentration (%, *w*/*v*) in brines (B) of Greek-style fermented cv. Konservolia black olives from the Magnesia (MAG) and Fthiotida (FTH) regions and of Spanish-style fermented cv. Halkidiki green olives from the Kavala (KAV) and Halkidiki (HAL) regions. Table S2A: Abundance of bacterial families identified in olive samples from the Magnesia (MAG), Fthiotida (FTH), Kavala (KAV), and Halkidiki (HAL) regions, Table S2B: Abundance of bacterial genera identified in olive samples from the Magnesia (MAG), Fthiotida (FTH), Kavala (KAV), and Halkidiki (HAL) regions, Table S2C: Abundance of bacterial species identified in olive samples from the Magnesia (MAG), Fthiotida (FTH), Kavala (KAV), and Halkidiki (HAL) regions, Table S3A: Abundance of yeast families identified in olive samples from the Magnesia (MAG), Fthiotida (FTH), Kavala (KAV), and Halkidiki (HAL) regions, Table S3B: Abundance of yeast genera identified in olive samples from the Magnesia (MAG), Fthiotida (FTH), Kavala (KAV), and Halkidiki (HAL) regions, Table S3C: Abundance of yeast species identified in olive samples from the Magnesia (MAG), Fthiotida (FTH), Kavala (KAV), and Halkidiki (HAL) regions.
